# Molecular alterations associated with pathophysiology in liver-specific ZO-1 and ZO-2 knockout mice

**DOI:** 10.1247/csf.24046

**Published:** 2024-09-26

**Authors:** Masahiko Itoh, Kenji Watanabe, Yoichi Mizukami, Hiroyuki Sugimoto

**Affiliations:** 1 Department of Biochemistry, School of Medicine, Dokkyo Medical University, Tochigi 321-0293, Japan; 2 Institute of Gene Research, Yamaguchi University Science Research Center, Yamaguchi 755-8505, Japan

**Keywords:** tight junctions, ZO-1/ZO-2 knockout mouse, liver, transcriptome analysis, molecular pathological progression

## Abstract

The liver is a complex organ with a highly organized structure in which tight junctions (TJs) play an important role in maintaining their function by regulating barrier properties and cellular polarity. Dysfunction of TJs is associated with liver diseases, including progressive familial intrahepatic cholestasis (PFIC). In this study, we investigated the molecular alterations in a liver-specific ZO-1 and ZO-2 double-knockout (DKO) mouse model, which exhibits features resembling those of PFIC4 patients with mutations in the ZO-2 gene. RNA-seq analysis revealed the upregulation of genes involved in the oxidative stress response, xenobiotic metabolism, and cholesterol metabolism in DKO livers. Conversely, the expression of genes regulated by HNF4α was lower in DKO livers than in the wild-type controls. Furthermore, age-associated analysis elucidated the timing and progression of these pathway changes as well as alterations in molecules related to TJs and apical polarity. Our research uncovered previously unknown implications of ZO-1 and ZO-2 in liver physiology and provides new insights into the molecular pathogenesis of PFIC4 and other tight junction-related liver diseases. These findings contribute to a better understanding of the complex mechanisms underlying liver function and dysfunction and may lead to the development of novel therapeutic strategies for liver diseases associated with tight junction impairment.

## Introduction

The liver is a multifunctional organ with a highly organized structure. Its ability to process nutrients, detoxify harmful substances, and produce bile is intricately linked to complex tissue architecture and cellular interactions. Hepatocytes, the main functional cells of the liver, are interconnected, creating a three-dimensional lattice that ensures efficient processing and transport within the liver. These cells are arranged in a radiating pattern from the periphery to the center of the lobule, forming a network known as the hepatic cord. Between adjacent hepatocytes lie bile canaliculi, channel-like structures that collect bile produced by hepatocytes (Arias *et al.*, 2020).

Tight junctions (TJs) are specialized structures found at the boundary of bile canaliculi in hepatocytes (Tsukita *et al.*, 2001). They act as barriers by regulating the passage of ions and molecules between cells, thereby maintaining distinct environments essential for cellular functions. The multiple protein complex, composed of transmembrane proteins, such as occludin and claudin families, and cytoplasmic proteins, such as ZO-1 (encoded by the *Tjp1* gene) and ZO-2 (encoded by the *Tjp2* gene), as well as the actin cytoskeleton and signaling molecules, constitutes TJs and regulates barrier functions (Zihni *et al.*, 2016). TJs are also vital for establishing and maintaining the polarity of hepatocytes, ensuring that different regions of the cell membrane have distinct functions such as the polarized transport of substances via specific transporters (Gissen and Arias, 2015). For example, the bile salt exporter Abcb11 and influx transporter Ntcp should be localized at the apical and basolateral membrane domains, respectively, and TJs are supposed to regulate the proper localization of these transporters.

Dysfunction of TJs is associated with several liver diseases, as it can lead to impaired barrier function and disrupted polarity (Pradhan-Sundd and Monga, 2019). This can result in the leakage of bile acids and other substances into the bloodstream, leading to cholestasis and further liver damage (Zollner and Trauner, 2008). Cholestasis can be caused by genetic mutations, such as those found in progressive familial intrahepatic cholestasis (PFIC) (Amirneni *et al.*, 2020). PFIC is a genetic disorder that results in impaired bile flow, leading to progressive liver disease and failure. Mutations in genes encoding bile transport proteins such as ATP8B1 (PFIC1) and ABCB11 (PFIC2), and tight junction protein ZO-2 (PFIC4), can disrupt bile flow regulation and contribute to cholestasis. All homozygous mutations in the ZO-2 gene in PFIC4 patients are predicted to inhibit protein translation, and liver specimens from these patients show almost undetectable ZO-2 protein expression (Sambrotta *et al.*, 2014). In addition, altered tight junction structures have been observed in hepatocytes of PFIC4 patients. These patients present with severe cholestasis and low GGT levels and often require liver transplantation in many cases. Therefore, elucidation of the detailed pathogenesis of PFIC4 at the molecular level is of great significance.

In a previous study, we generated liver-specific ZO-2 knockout mice to model the symptoms of PFIC4 patients. However, mutant mice did not exhibit cholestasis or altered tight junction structures. In contrast, when ZO-1, which has a similar primary structure and function to ZO-2, was inactivated simultaneously with ZO-2 in the liver, mutant double knockout (DKO) mice exhibited features resembling those of PFIC4 patients (Itoh *et al.*, 2021). In DKO mice, bilirubin and bile acids leaked into the blood, and serum ALT and ALP levels increased markedly, whereas GGT levels remained unchanged. Molecular and structural analyses revealed that TJs and cellular polarity, particularly apical polarity, were compromised in the DKO mouse livers. Furthermore, intravital imaging analysis confirmed that substances which should be secreted into the bile canaliculi from hepatocytes were instead secreted into sinusoidal blood vessels. These characteristics indicate that DKO mice can be used as a model of PFIC4; however, there is little information about the molecular alterations in DKO mice other than molecules implicated in TJs and cellular polarity.

In the present study, we employed RNA-seq analyses to understand the changes in the transcriptional landscape of the DKO mouse liver. We found that genes implicated in the oxidative stress response, xenobiotic metabolism, and cholesterol metabolism were upregulated in DKO livers. Conversely, the expression of genes regulated by HNF4α was lower in DKO livers than in the wild-type control livers. We also performed an age-associated analysis to determine when and how these pathways, as well as TJs and polarity, changed in DKO livers.

Our research revealed previously unknown implications of ZO-1 and ZO-2 in liver physiology, and provides new insights into the molecular pathogenesis of PFIC4 and other tight junction-related liver diseases.

## Materials and Methods

### Animals

The establishment and maintenance of the genetically modified mouse strains used in this study have been described previously (Itoh *et al.*, 2014; 2018; 2021) . Briefly, liver-specific ZO-1 and ZO-2 double-knockout (DKO) mice were generated by crossing *Tjp1^flox/flox^:Tjp2^flox/flox^* mice with *Alb-Cre*:*Tjp1^flox/+^:Tjp2^flox/+^* mice. All animal protocols were approved by the Committee of Care and Use of Laboratory Animals at Dokkyo Medical University (Permit Number: 06-517). All experiments were performed in accordance with the guidelines of Animal Research: Reporting of In Vivo Experiments. No more than five mice were housed in one cage and provided food and water ad libitum.

### Whole transcriptome analysis by RNA-seq

Livers from three WT and three DKO mice were utilized for transcriptome analysis. Total RNA was extracted from mouse liver using a Maxwell RNC simply RNA tissue kit (Promega Corp., Madison, WI, USA), and libraries were prepared as previously described (Kohno *et al.*, 2020). The library quality and concentration were assessed using a TapeStation 4200 (Agilent Technologies, Santa Clara, CA, USA). Libraries mixed with equal molecular concentrations were sequenced on an Illumina NextSeq500 DNA sequencer using a 75-bp paired-end cycle sequencing kit (Illumina Inc., San Diego, CA, USA). The data were then trimmed and mapped to the mouse GRCm38 release-94 reference genome using the CLC Genomics Workbench software (ver. 22.0.1; Qiagen Inc., Germantown, MD, USA, RRID:SCR_011853). The mapped read counts were normalized to transcripts per million (TPM) and converted to log2 after adding 1 to all TPM values. Principal component analysis (PCA) was conducted to assess variability in the RNA-seq data. The gene expression matrix was centered by subtracting the mean of each gene. The covariance matrix was then computed and eigen decomposition was performed to identify principal components. The centered data was projected onto these components to obtain PCA scores, which were used to visualize sample clustering and identify outliers. MA plots were generated to examine the relationship between gene expression levels and fold changes. Log2 fold changes (M values) were plotted against average log2 expression (A values) for each gene, providing a visualization of differential expression across conditions. Differentially expressed genes with p-value <0.05 and fold change >|2| were used for Ingenuity Pathway Analysis (Qiagen Inc., RRID: SCR_008653) and RNAseqChef analysis (Etoh and Nakao, 2023) to analyze the detected genes.

### Quantitative real-time PCR

Total RNA was isolated from mouse livers using the RNeasy Mini Kit (Qiagen Inc.) and reverse-transcribed using ReverTra Ace qPCR RT Master Mix (TOYOBO, Tokyo, Japan), according to the manufacturer’s instructions. Quantitative RT-PCR was performed on a QuantStudio 3 Real-Time PCR System (Thermo Fisher Scientific, Waltham, MA, USA) using the FastStart Universal SYBR Green Master (Roche Applied Science, Mannheim, Germany). For every gene in the age-associated analysis, the mRNA levels in each liver sample were normalized to β-actin, and the corresponding ratio was determined based on the value in the WT at P0, which was set as the reference point with a unit of measurement. All primers used for qRT-PCR are listed in Supplementary Table I.

### SDS-PAGE and western blot analysis

SDS-PAGE was performed using a standard protocol, and the separated proteins were transferred onto polyvinylidene fluoride (PVDF) membranes. The membranes were immersed in 5% skim milk dissolved in TBS for blocking and subsequently incubated with primary antibodies diluted in Immunoshot Reagent 1 (1:500) (Cosmo Bio, Tokyo, Japan). After being washed with TBS containing 0.2% Tween-20 (TBS-T), the membranes were incubated with HRP-conjugated secondary antibodies for mouse, rabbit, or rat Ig diluted in Immunoshot Reagent 2 (1:2,000). The membranes were then washed with TBS-T and incubated with Clarity Western ECL substrate (Bio-Rad). The signal was captured using the ChemiDoc system and quantified using the Image Lab software (Bio-Rad, Hercules, CA, USA).

### Immunostaining analysis

For immunostaining analysis, livers excised from the mice were embedded in OCT compound and frozen in liquid nitrogen. Cryosections were prepared from frozen liver tissue samples and fixed with 2% PFA for 15 min at room temperature or with cold ethanol for 10 min at –20°C. After washing with phosphate-buffered saline (PBS), the samples were blocked with 2% bovine serum albumin (BSA) in PBS and stained with primary antibodies diluted in Immunoshot Fine (1:100) (Cosmo Bio), followed by incubation with Alexa Fluor 594-conjugated secondary antibody diluted in Immunoshot Fine (1:300). Anti-Abcb11 rabbit polyclonal antibody and anti-4-HNE mouse monoclonal antibody were labeled utilizing Zenon Alexa Fluor 488 rabbit IgG Labeling kit (Z25303) and Zenon Alexa Fluor 594 Mouse IgG_1_ Labeling kit (Z25007) (Thermo Fisher Scientific), respectively. Stained samples were mounted in VectaShield Vibrance Antifade Mounting Medium with DAPI (H-1800, Vector Laboratories). Images were taken using a confocal microscope Zeiss LSM 710 (Zeiss) equipped with Blue Diode 405 (405 nm), Argon (458/488/514 nm), He-Ne 543 (543 nm) lasers and 4 objective lenses: 10× EC Plan-Neofluar (dry), 20× Plan-Apochromat (dry), 40× Plan-Apo (oil), 63× Alpha Plan-Apo(oil). Images processing was conducted using ZEN software (Zeiss), and quantitative analysis was performed using Fiji.

### Histological analysis

Histological analyses were performed as previously described (Itoh *et al.*, 2021). Briefly, liver samples were fixed with 20% formaldehyde solution, embedded in paraffin, and sectioned at 2-μm thickness. The sections were stained with hematoxylin and eosin, and images were captured with Olympus BX43 bright-field microscope (Olympus, Tokyo, Japan).

### Antibodies

Rabbit antibodies against Nqo1 (PA5-19624), ZO-3 (36-4100), claudin-3 (34-1700), occludin (71-1500) were obtained from Thermo Fisher Scientific; Srebp2 (28212-1-AP) and ZO-2 (18900-1-AP) from Proteintech (Rosemont, IL, USA), Abcb11 (PB9414) from Bosterbio (Pleasanton, CA, USA), Abcb1a (A19093), Ces3 (A4558), Cyp51 (A23370), Ephx1 (A2070), Hmgcr (A19063), HNF4α (A20865), and Ldlr (A14996) from ABclonal (Wuhan, China). Mouse monoclonal antibodies against Cyp2f2 (F-9), Cyp3a11 (P-6), and Gsta2 (E-6), and rat monoclonal antibody against ZO-1 (R40.76) were purchased from Santa Cruz Biotech (Dallas, TX, USA). Mouse monoclonal antibodies against β-actin (010-27841) and 4-HNE (300-15341) were purchased from FUJIFILM Wako (Tokyo, Japan). Alexa Fluor 594-conjugated anti-rabbit IgG (A32754) were purchased from Thermo Fisher Scientific. HRP-conjugated anti-mouse (7076), anti-rabbit (7074), and anti-rat (7077) IgG were purchased from Cell Signaling Technology (Danvers, MA, USA).

### Statistical analyses

For statistical analysis, the experiments were repeated three times unless stated otherwise, and the data are presented as the mean ± SEM. Statistical comparisons were performed between the wild-type control and double knockout groups. P-values were calculated using Student’s unpaired two-tailed t-test. Statistical significance was set at P<0.05.

## Results

### Upregulation of oxidative stress response and xenobiotic metabolism pathways in ZO-1 and ZO-2 deficient mouse liver

We previously found that approximately 50% and 80% of DKO mice die by 3 and 4 weeks of age, respectively, with severe cellular damage already observed in hepatocytes (Itoh *et al.*, 2021). On the other hand, DKO mice at 2 weeks of age began to show mortality, and the histological changes in the liver were not significant compared to those observed at 3 and 4 weeks of age (Fig. S1). Therefore, we considered the liver samples at this time point to be suitable for analyzing the molecular alterations associated with pathological progression in DKO livers.

**Fig. 1:**
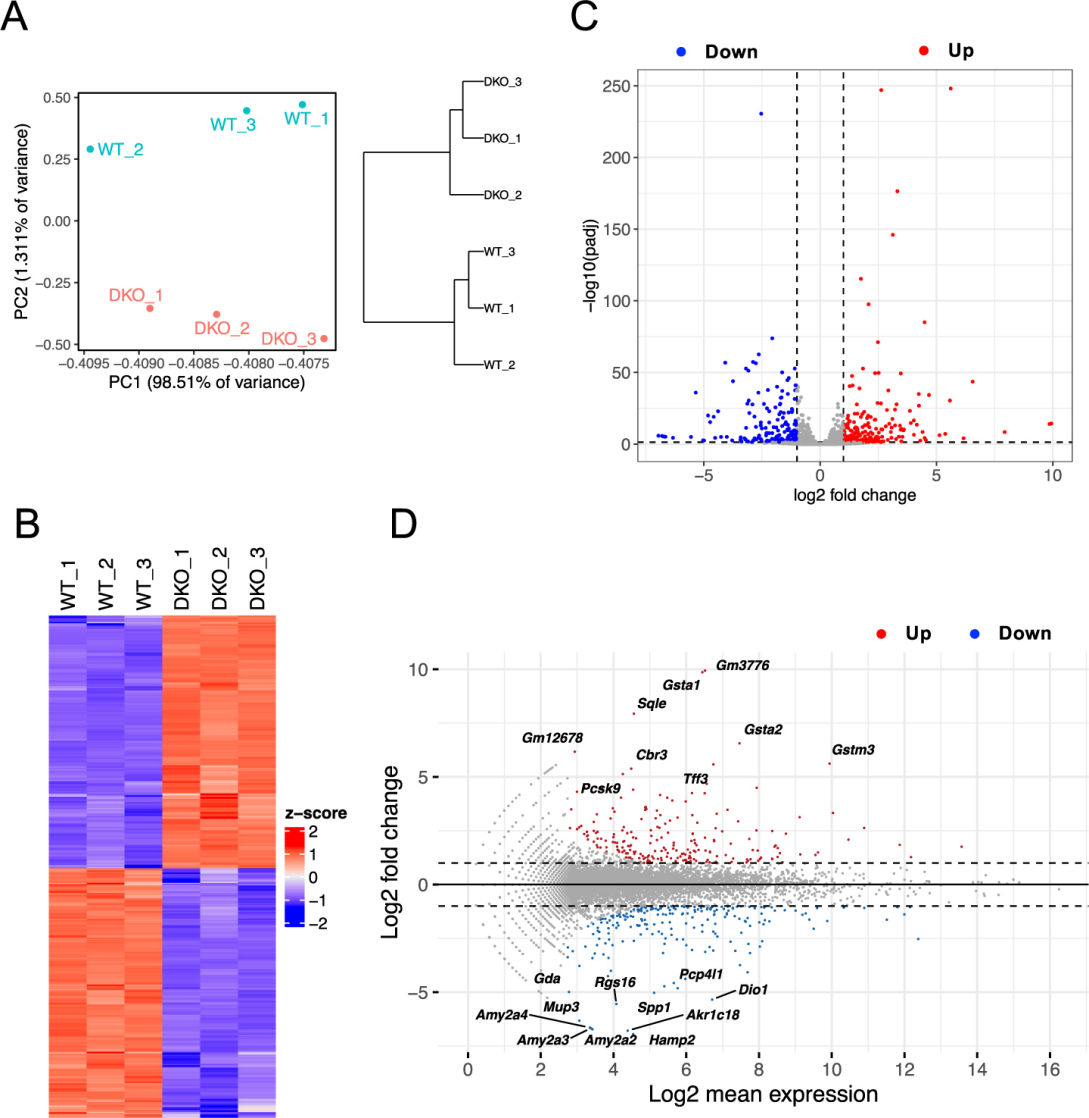
RNA-seq analysis showing differential gene expression in liver tissues of WT and DKO mice (A) PCA plots showing the clustering of samples based on gene expression profiles (left) and a dendrogram based on PCA (right). (B) Heat map illustrating the expression levels of differentially expressed genes across samples, with color gradients indicating high (red) to low (blue) expression levels. (C) Volcano plots displaying statistical significance (padj <0.05) versus fold-change of gene expression (fold change >|2|), highlighting significantly upregulated and downregulated genes. (D) MA plots showing the log-fold change against the mean expression for each gene, identifying differentially expressed genes in the dataset.

To comprehensively elucidate the molecules and pathways affected by the knockout of ZO-1 and ZO-2 in the liver, we extracted RNA from the livers of 2-week-old wild-type (WT) control and DKO mice, and conducted transcriptome analysis using RNA-seq. The expression profiling data were subjected to bioinformatic analysis. The PCA score plot revealed distinct clusters for WT and DKO liver samples (Fig. 1A). A heatmap depicted 431 genes that were differentially expressed and clustered into the WT and DKO groups (Fig. 1B). The volcano plot (Fig. 1C) and MA plot (Fig. 1D) indicated the presence of 217 upregulated genes (red dots) and 214 downregulated genes (blue dots) in DKO livers compared with WT livers. The top 50 upregulated (Table I) and downregulated (Table II) genes are shown. Pathway analysis was performed to gain biological insights into the transcriptomic alterations present in DKO livers, and a more detailed analysis was conducted.

**Fig. 2:**
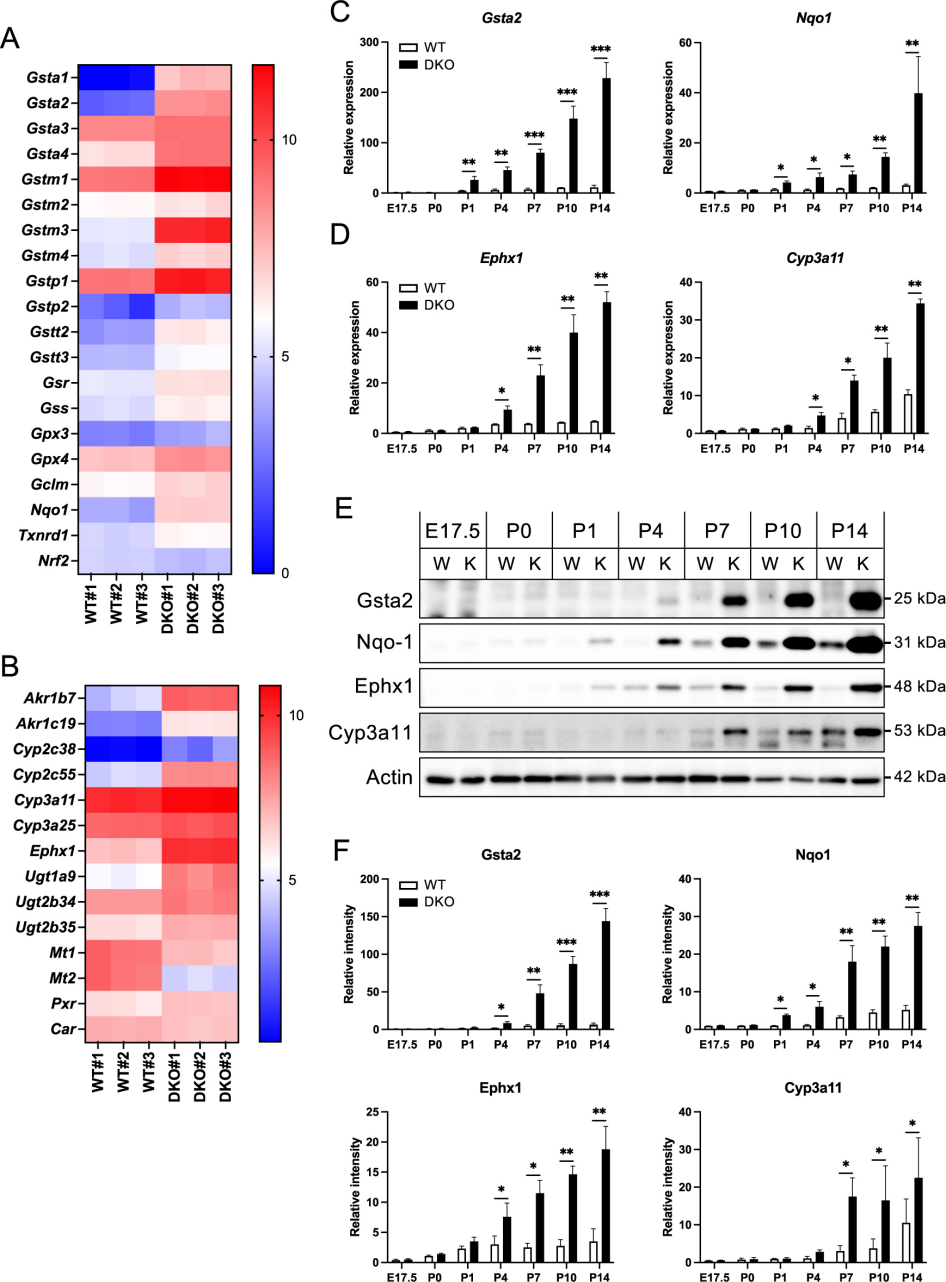
Expression of molecules implicated in oxidative stress response and xenobiotic metabolism pathways (A, B) Heat maps showing the differential expression of genes involved in the oxidative stress response (A) and xenobiotic metabolism pathways (B) in liver tissue from WT and DKO mice. (C, D) Quantification of relative mRNA levels of genes associated with the oxidative stress response (*Gsta2*, *Nqo1*) (C) and xenobiotic metabolism (*Eohx1*, *Cyp3a11*) (D) in WT and DKO livers from E17.5 to P14, as measured by qRT-PCR. β-Actin was used for normalization, with values in P0 WT set to 1 for comparison. Data are presented as the mean ± standard error of the mean (n = 3). Statistical significance relative to WT at each age is indicated as follows: *P<0.05, **P<0.01, and ***P<0.001. (E) Representative Western blot images showing the protein expression levels of Gsta2, Nqo1, Eohx1, and Cyp3a11 in WT and DKO livers from E17.5 to P14. β-Actin (Actin) was used as a loading control. W and K indicate WT and DKO, respectively. (F) The quantitative analysis of the western blot data. Data are presented as the mean ± standard error of the mean (n = 3). Statistical significance relative to WT at each age is indicated as follows: *P<0.05, **P<0.01, and ***P<0.001.

First, we found that the oxidative stress response pathway was upregulated in the DKO livers. Many genes associated with glutathione metabolism including family members of *Gsta*, *Gstm*, *Gstp*, *Gstt*, *Gpx* as well as *Gsr*, *Gss*, and *Gclm* were elevated in the DKO livers (Fig. 2A). This suggests an enhanced cellular effort to detoxify reactive oxygen species (ROS) via the glutathione pathway. Additionally, the transcription of other molecules that play a role in oxidative stress response, such as *Nqo1* and *Txnrd*1, was upregulated. These genes are known to be induced by Nrf2, a transcription factor that plays a crucial role in cellular defenses against oxidative stress (Ma, 2013). However, the mRNA level of *Nrf2* itself did not differ between WT and DKO livers.

To determine whether oxidative stress is indeed elevated in DKO livers, we assessed 4-hydroxynonenal (4-HNE), which is the primary by-product of lipid peroxidation and is widely utilized as an indicator of oxidative stress (Mooli *et al.*, 2022), in the liver sections by immunostaining with an antibody against 4-HNE (Fig. S2). In WT livers, 4-HNE was minimally detectable; conversely, abundant distinct 4-HNE signals were observed in DKO livers (Fig. S2A). Quantitative analysis of positive signals revealed a significant presence of 4-HNE in DKO livers (Fig. S2B), suggesting that oxidative stress was induced by the absence of ZO-1 and ZO-2 in the liver.

Generally, the xenobiotic metabolism pathway is activated in response to liver damage, and we found that phase I and phase II metabolism-related molecules were upregulated in DKO livers (Fig. 2B). In phase I, aldo-keto reductase (*Akr*) gene family detoxify and excrete xenobiotics by reducing the carbonyl groups (Penning, 2015). The cytochrome P450 (*Cyp*) 2c and 3a gene families are vital for the oxidative transformation of various xenobiotics and endogenous compounds (Esteves *et al.*, 2021). The molecule encoded by *Ephx1* gene hydrolyzes epoxides into less reactive dihydrodiols, thereby reducing cellular damage caused by reactive intermediates (Václavíková *et al.*, 2015). In phase II, the UDP-glucuronosyltransferase (*Ugt*) gene family, which is crucial for glucuronidation and excretion of diverse compounds (Oda *et al.*, 2015), was also upregulated in DKO livers. Pxr and Car transcription factors mainly regulate these molecules (Cai *et al.*, 2021). Pxr is activated by endogenous and exogenous compounds, such as steroids, bile acids, and drugs, whereas Car is activated by various ligands, including drugs and metabolites. A previous study indicated that Pxr or Car activation with agonists suppresses metallothionein (*Mt*)-1 and -2 transcription, which regulate and store essential metals such as zinc and copper (Cui and Klaassen, 2016). Our analysis revealed significantly lower *Mt-1* and *Mt-2* expression levels in DKO livers than in WT livers (Fig. 2B), indicating Pxr and/or Car activation in DKO livers.

### Analyzing molecular alterations associated with age

Next, we analyzed when and to what extent these molecular changes occurred. To this end, we first analyzed when the actual downregulation of ZO-1 and ZO-2 protein expression occurred. A previous study demonstrated that recombination by *Alb-Cre* in the liver begins in a mosaic-like manner around embryonic day (E) 15.5 (Weisend *et al.*, 2009). Therefore, we prepared protein lysates from the livers of *ZO-1/ZO-2* floxed mouse embryos at E15.5, with and without the *Alb-Cre* transgene. Western blotting analysis showed that the protein levels of ZO-1 and ZO-2 were almost the same in the livers of E15.5 mice of both genotypes (Fig. S3A, B). Next, we examined the liver at E17.5, and found that ZO-1 and ZO-2 protein expressions decreased in the presence of the *Alb-Cre* transgene. These results suggest that ZO-1 and ZO-2 expression begins to decline at E17.5, and that other molecules and pathways are affected at or later than E17.5.

To perform age-associated analysis, we prepared mRNA from the mouse liver at E17.5, postnatal day (P) 0, P1, P4, P7, P10, and P14 and conducted real-time qPCR assays. Among the oxidative stress response pathway, *Gsta2* mRNA levels did not significantly change with age in WT livers. In contrast, it increased at P1 and continued to increase thereafter, with a more than 100-fold increase at P14 compared to P0 in DKO livers (Fig. 2C). For *Nqo1*, WT livers showed a slight increase during the growth process, but a much larger increase was observed in DKO livers. As with *Gsta2*, the difference between WT and DKO livers for *Nqo1* expression was significant at P1. Next, we examined *Ephx1* and *Cyp3a11*, which are implicated in the xenobiotic metabolism pathway (Fig. 2D). Both genes showed a several-fold increase with age in WT livers, on the other hand, the elevation of those genes in DKO livers was much higher, and the difference between WT and DKO was evident at P4, indicating that xenobiotic metabolism pathway is activated later than oxidative stress response pathway.

Subsequently, we investigated the protein expression of these molecules (Fig. 2E, F). Western blotting analysis revealed that the Gsta2 protein was barely detectable in WT livers from E17.5 to P14, but a significant increase occurred from P4 to P7 and beyond in DKO livers. Nqo1 expression in WT livers exhibited a modest increase from E17.5 to P14. However, it demonstrated significantly elevated expression in DKO livers compared to WT livers at P1; subsequently, the disparity between WT and DKO livers progressively increased. In the case of xenobiotic molecules, a weak Ephx1 signal was detected in WT livers from P4 to P14 at approximately the same level, whereas explicit expression was detected in DKO livers around P1, with the expression level progressively increasing until P14. For Cyp3a11, minimal expression was observed at P7 in WT livers, and it increased to P14; conversely, DKO livers exhibited markedly higher protein expression than WT livers from P7 onward.

Overall, the age-associated alterations in the mRNA and protein levels are generally consistent for these pathways. The oxidative stress response pathway is activated approximately five days after the reduction of ZO-1 and ZO-2 protein expression in the liver, followed by the activation of the xenobiotic metabolism pathway a few days later.

### Srebp2 activity is enhanced in DKO livers

**Fig. 3:**
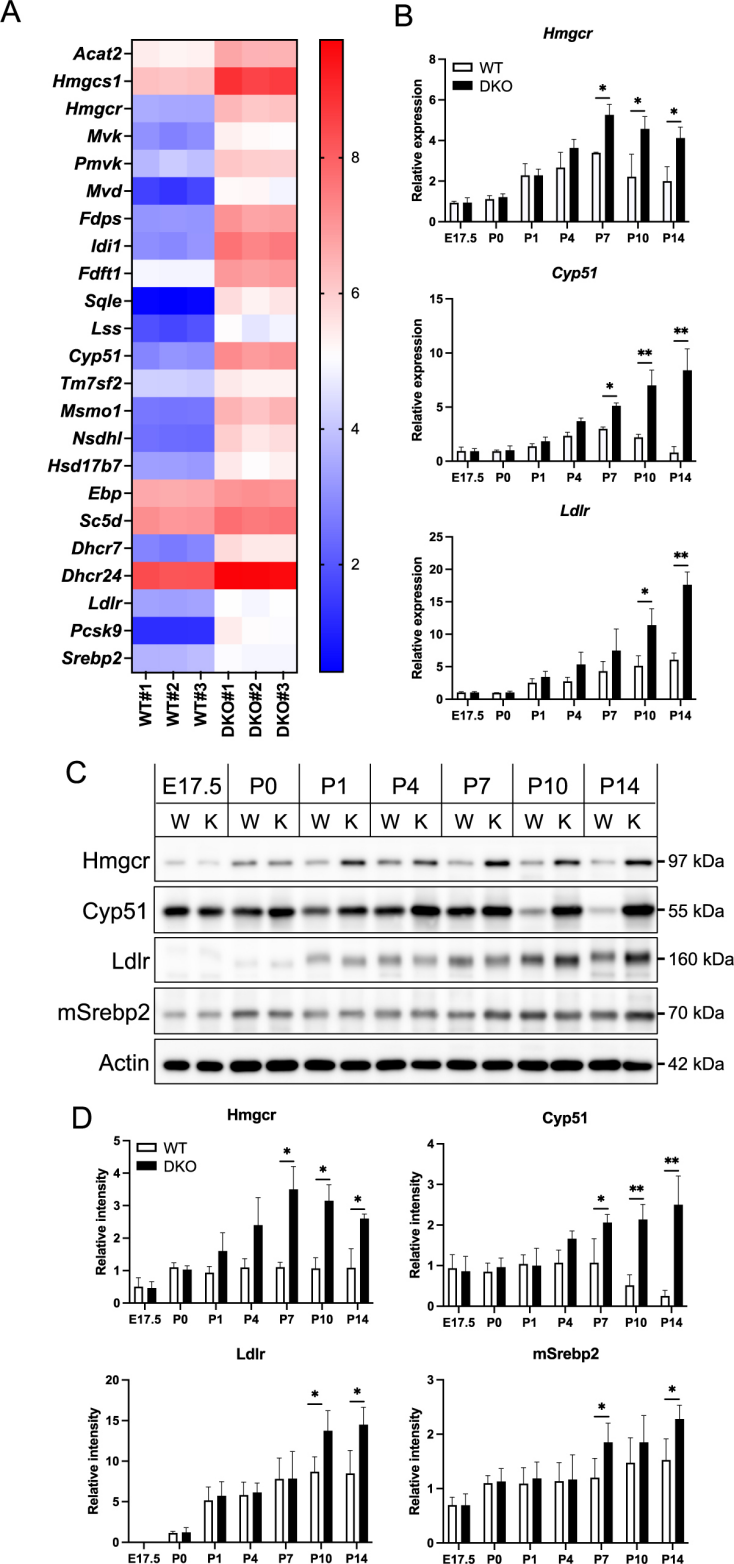
Differential expression of Srebp2-regulated molecules in liver tissues from WT and DKO mice (A) Heat map showing the differential expression of genes regulated by Srebp2 in WT and DKO livers. (B) Quantification of mRNA levels of Srebp2 target genes (Hmgcr, Cyp51, Ldlr) in WT and DKO livers from E17.5 to P14, as measured by qRT-PCR. β-Actin was used for normalization, with values in P0 WT set to 1 for comparison. Data are presented as mean ± standard error of the mean (n = 3). Statistical significance relative to WT is indicated as *P<0.05, **P<0.01. (C) Representative Western blot images showing the protein expression levels of Hmgcr, Cyp51, Ldlr, and mature Srebp2 (mSrebp2) in WT and DKO livers from E17.5 to P14. β-Actin (Actin) was used as a loading control. W and K indicate WT and DKO, respectively. (D) The quantitative analysis of the western blot data. Data are presented as mean ± standard error of the mean (n = 3). Statistical significance relative to WT is indicated as *P<0.05, **P<0.01.

Our transcriptome analysis revealed significant upregulation of the cholesterol biosynthesis pathway in DKO livers. The mRNA levels of most of the molecules involved in this process, such as *Hmgcr*, which initiates the production of mevalonate, and *Cyp51*, which contributes to the later stages of sterol formation (Brown *et al.*, 2021), were elevated in DKO livers (Fig. 3A).

These molecules are primarily transcriptionally activated by Srebp2, which is a crucial regulator of cholesterol homeostasis that controls the expression of genes involved not only in the biosynthesis but also in the uptake of cholesterol (Luo *et al.*, 2020). LDL receptors (Ldlr) primarily facilitate the uptake of cholesterol from the blood into hepatocytes, and Pcsk9 regulates the number of Ldlr molecules on the hepatocyte surface (Lagace, 2014). The expression of both *Ldlr* and *Pcsk9* genes was noticeably increased in DKO livers (Fig. 3A), suggesting that the transcriptional capacity of Srebp2 is higher in DKO livers compared to WT livers.

We performed age-associated expression analyses for *Hmgcr*, *Cyp51*, and *Ldlr*. In WT livers, the mRNA levels of *Hmgcr* and *Cyp51* showed a progressive increase from E17.5 to P7 and then declined slightly thereafter (Fig. 3B). In DKO livers, the levels of *Hmgcr* and *Cyp51* exhibited similar alterations up to P4, as those observed in WT livers. However, at P7, the levels of these genes were greater in DKO livers than in WT livers, and this difference continued to increase until P14. For *Ldlr*, mRNA levels increased several-fold with age in WT livers, while their elevation in DKO livers was substantially greater than in DKO livers at P10 and P14.

Subsequently, the protein expression analysis of these molecules was performed (Fig. 3C, D). Hmgcr and Cyp51 protein levels were higher in DKO livers than in WT livers around P7, with a more pronounced difference at P14. Although the difference in Ldlr protein levels between DKO and WT livers was less significant than that for Hmgcr and Cyp51, DKO livers still had higher amounts of Ldlr protein than WT livers, suggesting similar trends for alterations in protein levels with mRNA levels.

We also examined Srebp2 expression. Transcriptome data indicated slightly higher Srebp2 mRNA expression in DKO livers than in WT livers (Fig. 3A). Additionally, western blot analysis showed a subtle increase in the mature form of Srebp2 (mSrebp2) (Fig. 3C, D).

These results indicate that the cholesterol metabolism pathway is activated in DKO livers, while this activation occurs later than that of the oxidative stress response pathway and xenobiotic metabolism pathways, and that the dynamics of the temporal changes with age also differ from those of other pathways.

### Downregulation of HNF4α target molecules in DKO livers

**Fig. 4:**
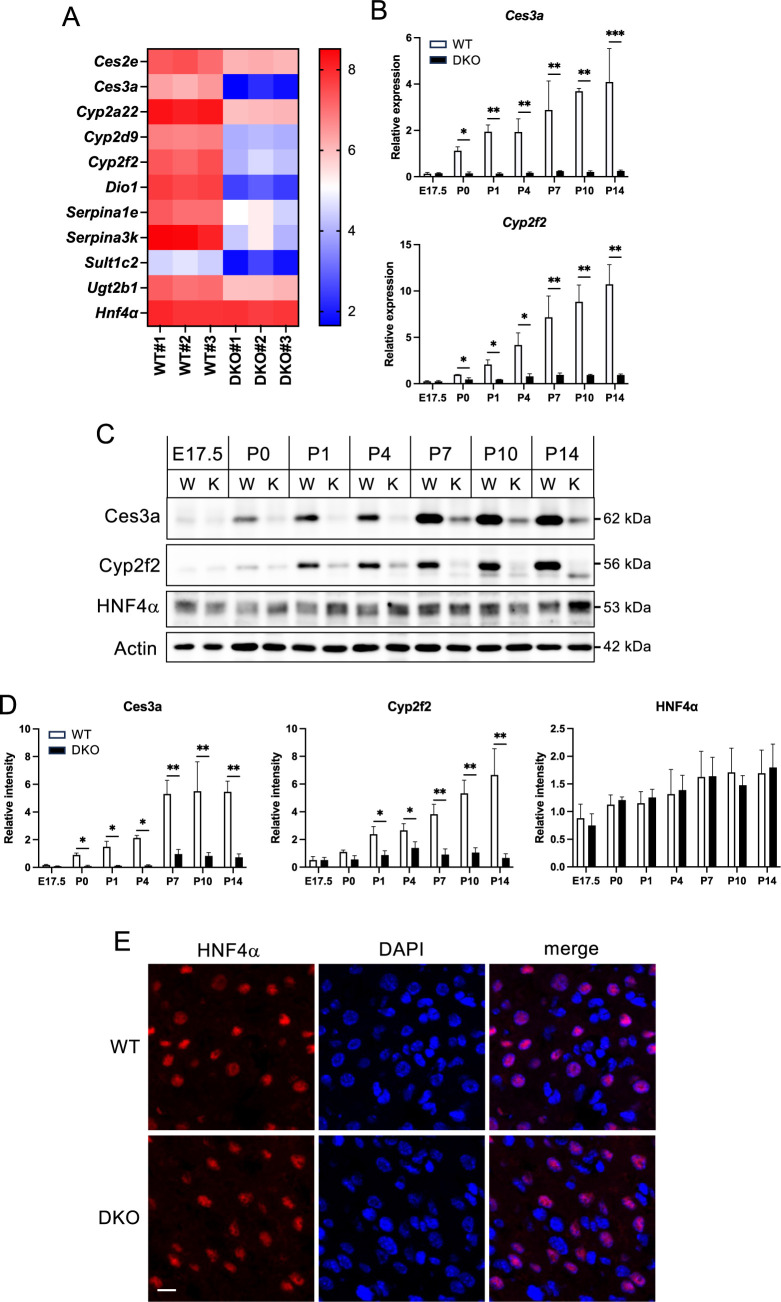
Expression of HNF4α and its target genes in WT and DKO livers (A) Heat map showing the differential expression of genes regulated by HNF4α in the WT and DKO livers. (B) Quantification of mRNA levels of HNF4α target genes (Ces3a, Cyp2f2) in WT and DKO livers from E17.5–P14 was performed using qRT-PCR. β-Actin was used for normalization, with values in P0 WT set to 1 for comparison. Data are presented as mean ± standard error of the mean (n = 3). Statistical significance relative to WT is indicated by *P<0.05, **P<0.01. (C) Representative Western blot images showing protein expression levels of Ces3a, Cyp2f2, Hmgcr, Cyp51, and HNF4α in WT and DKO livers from E17.5 to P14. β-Actin was used as a loading control. W and K indicate WT and DKO, respectively. (D) The quantitative analysis of the western blot data. Data are presented as mean ± standard error of the mean (n = 3). Statistical significance relative to WT is indicated as *P<0.05, **P<0.01. (E) Immunostaining images showing the localization and expression of HNF4α in WT and DKO livers. Sections were stained to visualize HNF4α protein distribution and intensity. Scale bar: 10 μm.

The classification of genes whose expression was downregulated in DKO livers was less clear than that of the upregulated genes. Nonetheless, a significant number of the downregulated genes (Fig. 4A) are regulated by HNF4α, a transcription factor that plays a critical role in promoting hepatocyte differentiation and maturation (Kotulkar *et al.*, 2023).

Carboxylesterases (Ces) are enzymes that hydrolyze esters and amides, with mature hepatocytes showing higher expression levels of *Ces* genes compared to immature or undifferentiated hepatocytes (Liu *et al.*, 2022). HNF4α regulates several *Cyp* family genes (Hwang-Verslues and Sladek, 2010) and directly influences *Dio1* expression, which is involved in thyroid hormone metabolism (Sinha *et al.*, 2018). HNF4α also controls *Sult1c2* expression, which is crucial for the metabolism of various endogenous compounds, including bile acids (Moscovitz and Aleksunes, 2013), and *Ugt* genes in the phase II metabolic pathways (Hwang-Verslues and Sladek, 2010). Additionally, HNF4α has been shown to regulate the transcription of several serine protease inhibitors such as *Serpina1e* and *Serpina3k* (Rollini and Fournier, 1999). These genes were downregulated in DKO livers, whereas the mRNA level of *Hnf4α* was unchanged (Fig. 4A).

In the course of analyzing mRNA expression in relation to age, it was observed that the levels of *Ces3a* and *Cyp2f2* exhibited a progressive and substantial increase in the livers of WT mice from E17.5 to P14. In contrast, the expression levels of these genes were only marginally elevated in the liver during the growth of the DKO mice (Fig. 4B).

Subsequently, the protein expression of Ces3a and Cyp2f2 was examined (Fig. 4C, D). The expression of both proteins exhibited a continuous increase in WT livers as maturation progressed. Conversely, their expression remained at low levels in DKO livers (Fig. 4C, D).

Overall, the age-associated alterations in the mRNA and protein levels of these molecules exhibited consistency, and the differential expression between DKO and WT livers manifested at an earlier stage compared to that of the Nrf2 target molecules (Fig. 2C–F).

We also determined protein levels of HNF4α in WT and DKO livers from E17.5 to P14 (Fig. 4C, D). HNF4α protein exhibited a modest increase with age in WT livers, and a comparable expression pattern was observed in DKO livers. In addition, we evaluated whether the nuclear localization of HNF4α was affected by ZO-1 and ZO-2 deficiency in the liver (Fig. 4E). However, no substantial differences were apparent in the localization patterns of HNF4α between the WT and DKO livers by immunostaining.

These findings imply that the downregulation of ZO-1 and ZO-2 protein expression in mouse liver from E17.5 onward may lead to a delay or suppression of hepatocyte differentiation and maturation, likely due to the dysregulation HNF4α transcriptional activity.

### Tight junction membrane protein dysregulation observed at P4

**Fig. 5:**
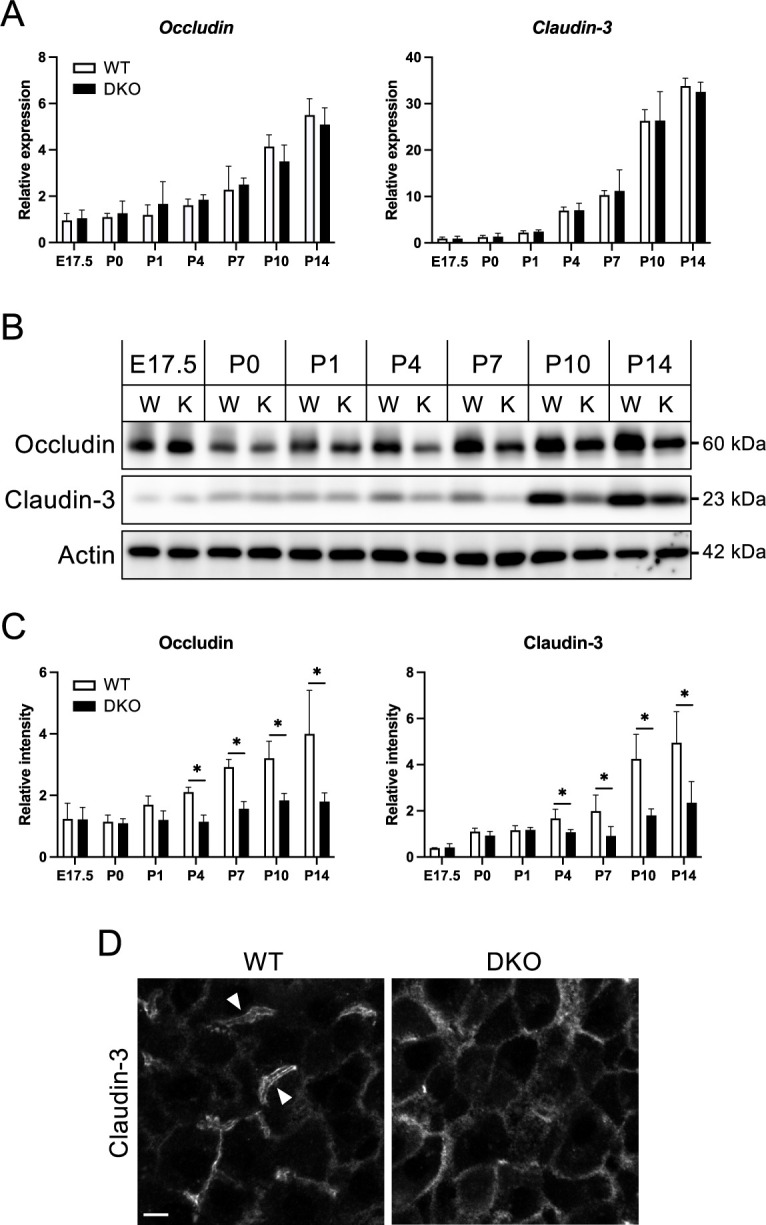
Expression and localization of tight junction membrane proteins during liver growth (A) Age-associated alterations in the mRNA levels of tight junction membrane proteins (occludin and claudin 3) from E17.5 to P14 were quantified by qRT-PCR. β-Actin was used for normalization. Data are presented as mean ± standard error of the mean (n = 3). (B) Western blot analysis of occludin and claudin 3 in the liver fractions, demonstrating protein expression levels. β-Actin was used as a loading control. W and K indicate WT and DKO, respectively. (C) The quantitative analysis of the western blot data. Data are presented as mean ± standard error of the mean (n = 3). Statistical significance relative to WT is indicated as *P<0.05. (D) Immunofluorescence microscopy of frozen WT and DKO liver sections at P4 was used to analyze the localization of claudin-3. Arrowheads indicate the tight junctions near the apical membrane domain in hepatocytes. Scale bar: 5 μm.

In a previous study, we showed that insoluble protein levels of TJ membrane proteins, such as occludin and claudin-3, were lower in DKO than in WT livers at 3 weeks of age, while mRNA levels were comparable (Itoh *et al.*, 2021). To determine the timing and extent of these differences, we performed age-associated qPCR and western blot analyses (Fig. 5A–C). Consistent with previous results, there were no differences in mRNA levels between WT and DKO livers at any age for either molecule (Fig. 5A). On the other hand, occludin and claudin-3 protein levels were reduced in DKO livers than in WT livers from P4 onward (Fig. 5B, C).

Then, the localization of claudin-3 in the liver at P4 was examined by immunofluorescence (Fig. 5D). In WT livers, claudin-3 exhibited a concentrated belt-like staining pattern, indicating the proper formation of TJs near the bile canaliculi in the apical region (Fig. 5D, arrowheads). In contrast, DKO livers lacked this apical enrichment, and claudin-3 was distributed diffusely across the entire plasma membrane, which was consistent with our previous observations of DKO livers at 3 weeks of age (Itoh *et al.*, 2021).

Based on the previous study demonstrating that ZO-1 and ZO-2 play a crucial role in the regulation of TJ barrier via modulating the localization of TJ membrane proteins in epithelial cells (Umeda *et al.*, 2006), our data suggest a potential involvement of ZO-1 and ZO-2 in establishing TJ barriers in hepatocytes around P4 during mouse liver growth.

### Differential regulation of apical transporters in DKO livers

**Fig. 6:**
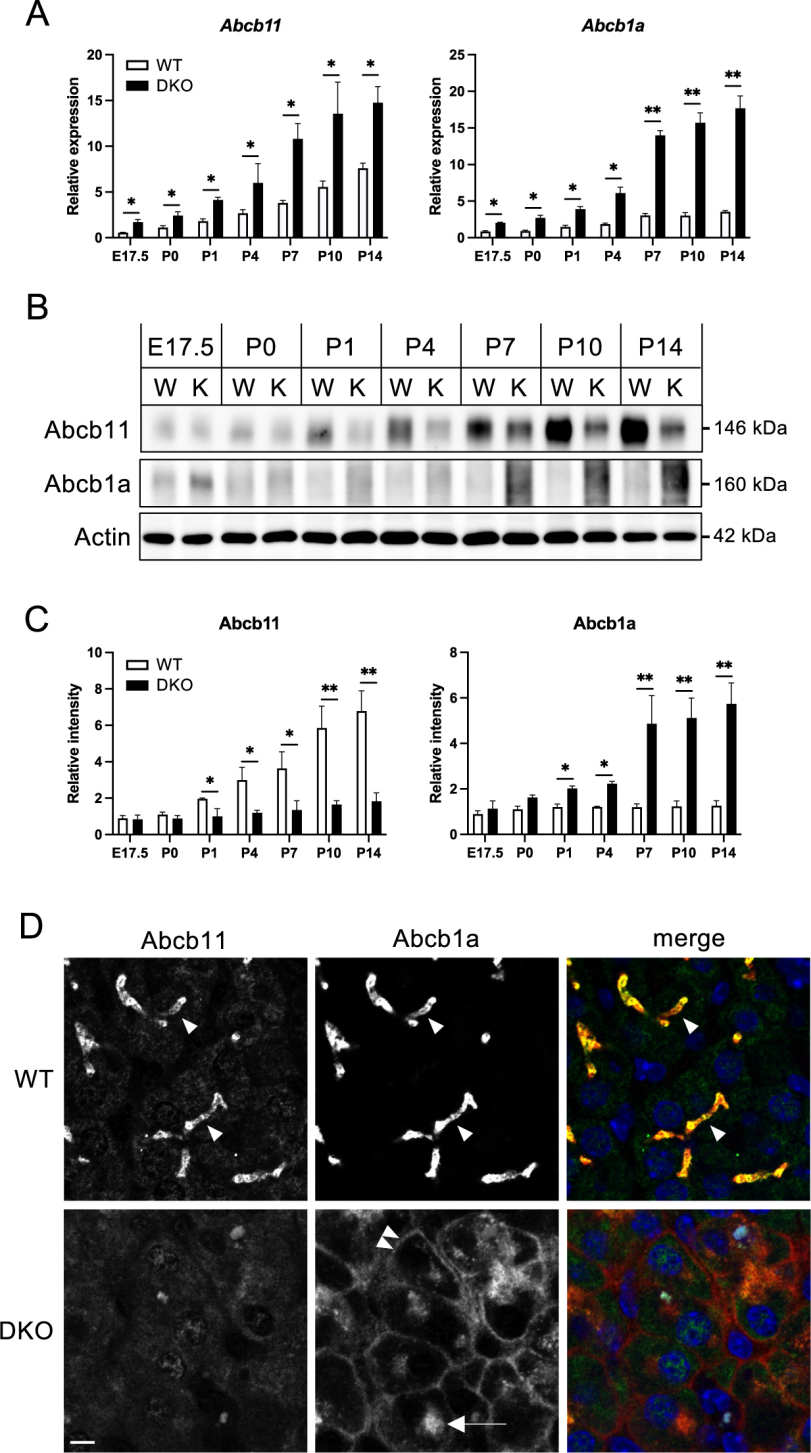
Expression and localization of Abcb11 and Abcb1a (A) Relative mRNA levels of apical membrane transporters Abcb11 and Abcb1a in WT and DKO livers from E17.5 to P4 were quantified by qRT-PCR. β-Actin was used for normalization. Data are presented as mean ± standard error of the mean (n = 3). Statistically significant differences from controls are indicated by *P<0.05, **P<0.01. (B) Western blot analysis of Abcb11 and Abcb1a in total liver lysates, showing protein expression levels in WT and DKO livers from E17.5 to P14. β-Actin was used as a loading control. W and K indicate WT and DKO, respectively. (C) The quantitative analysis of the western blot data. Data are presented as mean ± standard error of the mean (n = 3). Statistical significance relative to WT is indicated as *P<0.05, **P<0.01. (D) Immunofluorescence microscopy of frozen liver sections from WT and DKO mice at P4 was used to examine the localization of Abcb11 and Abcb1a. The arrowheads in the WT images indicate bile canalicular apical membrane domains. In DKO livers stained for Abcb1a, the signal was detected all-around plasma membrane (double-arrowhead) and in an intracellular dot-like structure (arrow). Scale bar: 5 μm.

Our previous study demonstrated that cell polarity, especially apical polarity, is impaired in DKO hepatocytes, resulting in the altered localization and expression of apical efflux transporters, such as Abcb11. Furthermore, in vivo imaging analysis showed a significant reduction in the secretion of substances from hepatocytes into bile canaliculi (Itoh *et al.*, 2021). To determine when the alterations of apical transporters such as Abcb11 and Abcb1a occur in DKO livers, we conducted an age-associated analysis (Fig. 6).

In both WT and DKO livers, the mRNA levels of *Abcb11* and *Abcb1a* increased progressively from E17.5 to P14. However, the degree of increase was significantly higher in DKO livers than in WT livers (Fig. 6A). Notably, the differences between the WT and DKO livers were evident even at E17.5, when ZO-1 and ZO-2 protein levels were reduced in the liver (Fig. S3). Subsequently, we conducted a study to investigate the protein expression levels of these molecules (Fig. 6B, C). In WT livers, Abcb11 protein levels increased progressively with age, consistent with the increase in mRNA levels (Fig. 6A). However, in DKO livers, Abcb11 protein expression did not exhibit a marked increase with age, which was inconsistent with the changes in mRNA levels. In contrast, Abcb1a protein expression mirrored mRNA changes in both WT and DKO livers, with higher levels in DKO than in WT from E17.5 and widening differences with age (Fig. 6B, C).

It has been shown that the localization of both Abcb11 and Abcb1a in the bile canalicular membrane in hepatocytes is regulated through the apical recycling endosome (Gissen and Arias, 2015). Immunostaining analysis showed that WT livers exhibited co-localization of Abcb11 and Abcb1a at the bile canalicular apical membrane domains (Fig. 6D, arrowheads). In contrast, DKO livers exhibited weak and diffuse staining for Abcb11, which was consistent with our previous observations (Itoh *et al.*, 2021). Conversely, Abcb1a was detected along the entire plasma membrane (Fig. 6D, double-arrowhead) and within an intracellular dot-like structure in DKO livers (Fig. 6D, arrow). These data suggest that the intracellular transport pathways for Abcb11 and Abcb1a might be differentially regulated with the involvement of ZO-1 and ZO-2, and the loss of these ZO molecules leads to improper expression and/or localization of apical transporters, which could impair the liver physiology.

## Discussion

In the present study, we newly discovered that the suppression of ZO-1 and ZO-2 in the liver from E17.5 is associated with the activation of Nrf2, Pxr, and Car. Nrf2 activation leads to the upregulation of antioxidant proteins such as Gst and Nqo1 (Zhou *et al.*, 2022). These proteins help to neutralize ROS and mitigate oxidative damage. Pxr and Car regulate the expression of genes involved in drug metabolism, detoxification, and energy homeostasis (Cai *et al.*, 2021). Generally, oxidative stress and metabolic abnormalities can cause mitochondrial swelling and disruption of the internal membrane structures (Cheville, 2013). Additionally, the appearance of vacuoles within cells is considered part of the cellular response to oxidative stress. Furthermore, an imbalance in intracellular water and ions can cause cell swelling (Gusev and Zotova, 2019). All of these characteristics were observed in the livers of DKO mice at 3 weeks of age in our previous work (Itoh *et al.*, 2021), and the data presented in this paper support the hypothesis that these structural abnormalities result from oxidative stress and metabolic abnormalities.

Additionally, age-associated analysis revealed that the oxidative stress response pathway is activated as early as P1. This may be related to the significant increase in bile production and secretion after birth. In humans, cholestasis is known to enhance oxidative stress (Copple *et al.*, 2010), and it is possible that cholestasis similarly causes oxidative stress in DKO livers, leading to the activation of the Nrf2 pathway at P1. In contrast, the transcriptional upregulation of Pxr and Car target genes occurred at P4, later than that of the Nrf2 targets. This may be consistent with a previous report that the activation of the Nrf2 pathway can enhance the activity of Pxr and Car (Ashino *et al.*, 2020). Conversely, ROS are produced during metabolism by detoxifying enzymes that are regulated by Pxr and Car (Willson and Kliewer, 2002), which can lead to persistent oxidative stress. In any case, the pathways that protect hepatocytes from damage are activated soon after the suppression of ZO-1 and ZO-2; however, their capability is probably insufficient.

The cholesterol metabolism pathway regulated by Srebp2 is another molecular pathway that is upregulated in DKO livers. Previous studies have shown that oxidative stress and Pxr activation stimulate Srebp2 (Karpale *et al.*, 2021; Seo and Shin, 2017). , and our findings that the difference in the expression of Srebp2 target genes between WT and DKO livers occurs after that of Nrf2 or Pxr targets, suggesting that oxidative stress and Pxr activation may contribute to Srebp2 activation in DKO livers. Srebp2 is synthesized as an inactive precursor protein bound to the ER membrane, forming a complex with Scap. The Srebp2-Scap complex is transported from the ER to the Golgi apparatus, where Srebp2 undergoes two proteolytic cleavage steps. Through this process, the N-terminal domain of Srebp2, which is the mature form and functions as a transcription factor, is released from the Golgi apparatus and translocated to the nucleus (Shimano and Sato, 2017). The protein level of mature Srebp2 in DKO livers was only slightly higher than that in WT livers, leaving it unclear whether this is sufficient to explain the upregulation of Srebp2 target genes in DKO livers. Due to issues with the antibodies used, we were unable to obtain data on the intracellular localization of Srebp2 in this study. If we can clarify Srebp2 localization in future research, we may gain new insights into the molecular mechanisms underlying Srebp2 activation and cholesterol metabolism in DKO livers.

Meanwhile, there are few liver-related molecular pathways whose expression levels are clearly lower in DKO than in WT livers, and the obvious ones are a group of molecules whose expression is regulated by HNF4α. HNF4α is a key transcription factor in the liver, influencing both its differentiation during development and its function in adult liver tissue (Kotulkar *et al.*, 2023). We found that HNF4α itself was not differentially expressed between WT and DKO livers at the mRNA and protein levels, nor was its subcellular localization pattern affected in DKO livers, suggesting that another factor suppresses HNF4α activation in DKO livers. Previous studies have shown that persistent inflammation or fibrosis suppresses HNF4α function (Vető *et al.*, 2017); however, this does not seem to be the reason in the case of DKO livers, since no inflammatory lesions were observed and no elevation of inflammatory cytokines like IL-1β occurred in DKO livers in our previous study (Itoh *et al.*, 2021). It has also been suggested that ROS can reduce the DNA-binding capacity and transcriptional activity of HNF4α (Vető *et al.*, 2017), which may explain the lack of upregulation of HNF4α target gene expression in DKO livers. In age-associated analysis, the expression of HNF4α target genes continued to increase from P0 in WT livers. On the other hand, there was little increase in DKO livers, showing a difference from WT as early as P0, which occurs before the alterations in the Nrf2-dependent oxidative stress response genes appear. It is possible that HNF4α is affected by lower levels of ROS than those required to influence Nrf2 activity. HNF4α dysfunction has been suggested to impair the metabolic function of hepatocytes (Sang *et al.*, 2022), potentially leading to mitochondrial dysfunction and oxidative stress. Furthermore, HNF4α is involved in the expression of some detoxifying enzymes (Hwang-Verslues and Sladek, 2010), and its dysfunction can reduce the expression of these enzymes, resulting in the accumulation of harmful substances and metabolic byproducts, which can also increase oxidative stress.

We previously reported structural and functional abnormalities in the TJs and apical polarity in DKO livers (Itoh *et al.*, 2021). In this study, we found that alterations in TJ membrane proteins begin at P4 without changes in mRNA levels, whereas changes in apical transporters such as Abcb11 and Abcb1a occur at embryonic E17.5, accompanied by an increase in mRNA levels. Abcb11 is known to be transcriptionally regulated by the farnesoid X receptor (FXR) (Mazuy *et al.*, 2015), but many other FXR-regulated molecules, such as Cyp7a1 and Cyp8b1, are not upregulated in DKO livers. Therefore, the higher Abcb11 mRNA levels in DKO livers cannot be solely attributed to enhanced FXR activity. On the other hand, it has been shown that the transcription of Abcb1a is activated by Pxr (Jonker *et al.*, 2012); however, most Pxr-regulated molecules exhibited transcriptional differences between WT and DKO livers starting from P4, suggesting that the increased transcription of Abcb1a is not merely due to the activation of Pxr.

Protein expression analysis revealed lower Abcb11 levels in DKO livers than in WT livers, despite the higher mRNA levels. In contrast, Abcb1a protein levels were higher in DKO livers, consistent with the mRNA levels, suggesting the implication of distinct post-translational regulation. Our immunostaining analysis of DKO livers demonstrated weak signals for Abcb11, whereas Abcb1a was distributed across the entire plasma membrane and intracellular dot-like structures. This suggests the existence of multiple transport pathways within the ARE and/or distinct protein stabilities, with one pathway involving Abcb11 leading to degradation when proper transport fails and another involving Abcb1a leading to disorganized transport to the cell membrane and an increased protein amount. It is possible that these changes in bile canalicular apical transporter expression and localization induce oxidative stress and affect the liver function. Further analyses are needed to elucidate the molecular mechanisms underlying apical transporter localization and protein stability in hepatocytes in relation to ZO-1 and ZO-2.

In summary, our research revealed that the suppression of ZO-1 and ZO-2 in hepatocytes from E17.5 affects metabolic and differentiation pathways in the liver. ZO-1 and ZO-2 have been shown to function as signaling molecules in addition to their role in regulating the TJ barrier (Balda *et al.*, 2003; Itoh *et al.*, 2012; Ouyang *et al.*, 2023). The altered expression of various molecules in DKO livers may thus be attributed to defects in the signaling mechanisms mediated by ZO-1 and ZO-2. In addition, impaired bile acid excretion due to the mis-localization of the bile acid transporter Abcb11 could lead to oxidative stress, which in turn may induce changes in the expression of various genes. Similarly, the mis-localization of Abcb1a, an efflux pump responsible for the active transport of a wide range of xenobiotics, could increase the expression of xenobiotic response molecules in hepatocytes. Given that the oxidative stress response appears to occur early after birth, administering ROS-neutralizing substances, such as vitamin E and the antioxidant N-acetylcysteine, to pregnant mother mice could mitigate the phenotypes observed in DKO mice and potentially extend their lifespan. Future studies utilizing our mouse model could provide valuable insights into the understating of the liver pathophysiology associated with the suppression of ZO-1 and ZO-2, thereby facilitating the development of more efficacious therapeutic interventions for PFIC4 patients.

## Figures and Tables

**Table I:** Top 50 genes up-regulated in DKO mouse liver

Gene Symbol	Gene Description	Difference	Mean of WT	Mean of DKO	q value
*Gsta1*	glutathione S-transferase, alpha 1	7.526	0.184	7.71	0.001104
*Gm3776*	predicted gene 3776	7.129	0	7.129	0.000796
*Gsta2*	glutathione S-transferase, alpha 2	6.02	2.45	8.47	0.000796
*Gstm3*	glutathione S-transferase, mu 3	5.588	5.437	11.02	0.000796
*Sqle*	squalene epoxidase	4.941	0.5923	5.533	0.0012
*Tff3*	trefoil factor 3	4.932	2.955	7.887	0.003982
*Akr1b7*	aldo-keto reductase family 1, member B7	4.423	4.434	8.857	0.001927
*Idi1*	isopentenyl-diphosphate delta isomerase	4.381	3.047	7.428	0.001104
*Cbr3*	carbonyl reductase 3	4.379	1.243	5.622	0.000796
*Cyp51*	cytochrome P450, family 51	4.172	2.837	7.009	0.000796
*Ugdh*	UDP-glucose dehydrogenase	4.04	3.349	7.389	0.000796
*Ces2c*	carboxylesterase 2C	3.979	2.256	6.235	0.004304
*Msmo1*	methylsterol monoxygenase 1	3.885	2.33	6.216	0.001456
*Fdps*	farnesyl diphosphate synthetase	3.855	3.077	6.933	0.00112
*Pcsk9*	proprotein convertase subtilisin/kexin type 9	3.854	1.465	5.319	0.001433
*Olig1*	oligodendrocyte transcription factor 1	3.807	1.792	5.6	0.001472
*Gm12678*	predicted gene 12678	3.733	0.1511	3.884	0.001761
*Scd2*	stearoyl-Coenzyme A desaturase 2	3.729	2.667	6.396	0.00697
*Mvd*	mevalonate decarboxylase	3.696	1.57	5.266	0.001104
*Slc51b*	solute carrier family 51, beta subunit	3.547	3.132	6.679	0.003142
*Rasal1*	RAS protein activator like 1	3.507	0.7182	4.225	0.002313
*9130409I23Rik*	delta 4-desaturase, sphingolipid 1 like	3.499	1.061	4.561	0.001853
*Bex1*	brain expressed X-linked 1	3.374	2.385	5.759	0.004343
*Igf2*	insulin-like growth factor 2	3.341	7.74	11.08	0.001749
*Cacna1b*	calcium channel, voltage-dependent, N type, alpha 1B	3.326	0.6325	3.958	0.001472
*Cyp2c55*	cytochrome P450, family 2, subfamily c, polypeptide 55	3.319	4.56	7.879	0.001433
*Krt20*	keratin 20	3.315	2.318	5.633	0.003681
*Smoc2*	SPARC related modular calcium binding 2	3.308	2.404	5.712	0.001143
*Baiap2l2*	BAI1-associated protein 2-like 2	3.299	0.1913	3.491	0.001565
*Akr1c19*	aldo-keto reductase family 1, member C19	3.283	2.714	5.997	0.000796
*Hist1h2ba*	H2B clustered histone 1	3.269	0	3.269	0.000796
*Amn*	amnionless	3.25	0.54	3.79	0.001693
*Nsdhl*	NAD(P) dependent steroid dehydrogenase-like	3.248	2.423	5.672	0.001643
*Nqo1*	NAD(P)H dehydrogenase, quinone 1	3.224	3.775	7	0.002957
*Wfdc3*	WAP four-disulfide core domain 3	3.201	0.4064	3.608	0.002957
*Acot3*	acyl-CoA thioesterase 3	3.192	2.995	6.187	0.001393
*Scd1*	stearoyl-Coenzyme A desaturase 1	3.167	4.447	7.614	0.00528
*Ephx1*	epoxide hydrolase 1, microsomal	3.134	6.961	10.09	0.000796
*Phlda2*	pleckstrin homology like domain, family A, member 2	3.124	0.1473	3.271	0.003105
*Tuba8*	tubulin alpha 8	3.093	1.843	4.936	0.002337
*Ugt1a9*	UDP glucuronosyltransferase 1 family, polypeptide A9	2.964	5.197	8.161	0.002957
*Lss*	lanosterol synthase	2.946	1.959	4.905	0.003105
*Hnf4aos*	hepatic nuclear factor 4 alpha, opposite strand	2.905	0.9062	3.811	0.007087
*Tsku*	tsukushi, small leucine rich proteoglycan	2.879	3.222	6.1	0.000796
*Igdcc4*	immunoglobulin superfamily DCC subclass member 4	2.821	0.3623	3.183	0.001965
*Gstt2*	glutathione S-transferase, theta 2	2.818	3.626	6.444	0.002827
*Srxn1*	sulfiredoxin 1	2.816	4.384	7.2	0.000796
*Tfap2e*	transcription factor AP-2, epsilon	2.804	0	2.804	0.001222
*Dhcr7*	7-dehydrocholesterol reductase	2.781	2.965	5.746	0.001565
*Slc4a9*	solute carrier family 4, sodium bicarbonate cotransporter, member 9	2.778	0.6194	3.397	0.01099

**Table II:** Top 50 genes down-regulated in DKO mouse liver

Gene Symbol	Gene Description	Difference	Mean of WT	Mean of DKO	q value
*Dio1*	deiodinase, iodothyronine, type I	–5.048	7.663	2.615	0.001188
*Spp1*	secreted phosphoprotein 1	–4.893	6.021	1.128	0.033315
*Hamp2*	hepcidin antimicrobial peptide 2	–4.875	5.32	0.4448	0.015219
*Akr1c18*	aldo-keto reductase family 1, member C18	–4.777	5.229	0.4527	0.009806
*Pcp4l1*	Purkinje cell protein 4-like 1	–4.465	6.694	2.229	0.003674
*Ces3a*	carboxylesterase 3A	–4.407	6.32	1.913	0.002273
*Amy2a2*	amylase 2a2	–4.292	4.326	0.03428	0.000288
*Rgs16*	regulator of G-protein signaling 16	–4.242	4.979	0.7371	0.002748
*Amy2a4*	amylase 2a4	–4.227	4.271	0.044	0.000588
*Amy2a3*	amylase 2a3	–4.216	4.289	0.07324	0.000887
*Cyp4f14*	cytochrome P450, family 4, subfamily f, polypeptide 14	–4.193	6.573	2.38	0.002138
*Cyp3a44*	cytochrome P450, family 3, subfamily a, polypeptide 44	–4.115	6.878	2.763	0.001829
*Mt2*	metallothionein 2	–4.01	8.572	4.562	0.003077
*Mup3*	major urinary protein 3	–3.823	3.968	0.1449	0.002309
*Serpina3k*	serine (or cysteine) peptidase inhibitor, clade A, member 3K	–3.775	8.352	4.577	0.011262
*Fmo3*	flavin containing monooxygenase 3	–3.611	4.731	1.119	0.001377
*Raet1d*	retinoic acid early transcript delta	–3.553	4.571	1.018	0.000895
*Gck*	glucokinase	–3.462	4.739	1.276	0.007115
*Selenbp2*	selenium binding protein 2	–3.141	8.581	5.44	0.003106
*B3galt1*	beta 1,3-galactosyltransferase, polypeptide 1	–3.105	4.688	1.582	0.003222
*Cyp2f2*	cytochrome P450, family 2, subfamily f, polypeptide 2	–3.07	7.346	4.276	0.003422
*Akr1c20*	aldo-keto reductase family 1, member C20	–3.039	8.176	5.137	0.000398
*Acnat2*	acyl-coenzyme A amino acid N-acyltransferase 2	–2.998	6.142	3.144	0.008645
*Mug1*	murinoglobulin 1	–2.989	7.552	4.564	0.002735
*Gm12909*	predicted gene 12909	–2.97	2.97	0	0.007115
*Gda*	guanine deaminase	–2.938	3.57	0.6318	0.001972
*Nrep*	neuronal regeneration related protein	–2.938	6.975	4.037	0.003562
*Car3*	carbonic anhydrase 3	–2.88	8.827	5.947	0.005454
*Sult2a8*	sulfotransferase family 2A, member 8	–2.865	7.441	4.577	0.005899
*Hopx*	HOP homeobox	–2.806	4.244	1.438	0.004017
*Cyp2d9*	cytochrome P450, family 2, subfamily d, polypeptide 9	–2.753	6.794	4.041	0.000552
*1700080G11Rik*	RIKEN cDNA 1700080G11 gene	–2.75	2.75	0	0.005409
*Cyp3a16*	cytochrome P450, family 3, subfamily a, polypeptide 16	–2.732	8.499	5.766	0.00207
*Ngp*	neutrophilic granule protein	–2.714	4.183	1.469	0.015037
*Spry4*	sprouty homolog 4 (Drosophila)	–2.707	3.851	1.143	0.000895
*Rgs5*	regulator of G-protein signaling 5	–2.693	4.705	2.012	0.02712
*Tbx3os1*	T-box 3, opposite strand 1	–2.65	2.935	0.2847	0.007899
*C9*	complement component 9	–2.638	6.176	3.538	0.001801
*Apon*	apolipoprotein N	–2.629	7.994	5.364	0.004733
*Cyp1a2*	cytochrome P450, family 1, subfamily a, polypeptide 2	–2.628	8.863	6.235	0.00305
*Slc22a29*	solute carrier family 22. member 29	–2.535	5.003	2.468	0.029031
*Chac1*	ChaC, cation transport regulator 1	–2.523	2.995	0.4719	0.002435
*mt-Rnr1*	12S rRNA, mitochondrial	–2.518	13.14	10.62	0.000856
*Slc22a27*	solute carrier family 22. member 27	–2.496	6.667	4.171	0.008133
*Gm20388*	Polypeptide N-Acetylgalactosaminyltransferase 2	–2.474	3.421	0.9473	0.176852
*Clec2d*	C-type lectin domain family 2, member d	–2.474	6.495	4.02	0.007508
*Sult1c2*	sulfotransferase family, cytosolic, 1C, member 2	–2.466	4.532	2.066	0.010877
*Gm45774*	predicted gene 45774	–2.437	3.491	1.055	0.007926
*Inhbe*	inhibin beta-E	–2.433	3.633	1.2	0.005751
*Rarres1*	retinoic acid receptor responder 1	–2.41	5.308	2.898	0.004734
