# Supplementary material for: Molecular alterations associated with pathophysiology in liver-specific ZO-1 and ZO-2 knockout mice
Source: Cell Struct Funct. 2024 Sep 26;49(2):83–99. doi: 10.1247/csf.24046 (PMC11930773; doi:10.1247/csf.24046)
Supplement: Supplementary file 1 — Supplementary Materials [file csf_49_24046_1.zip › 49_24046_Suppl_Fig_legends/49_24046_Supplementary_Table_I.pdf]

Supplementary Table I. qPCR primer sequences

Gene	Forward (5'-3')	Reverse (5'-3')
Abcb11	GGACACACCTGAGAGGACCTT	AGATCGTTGACGGATGGAAG
Abcb1a	GGGCATTTACTTCAAACTTGTCA	TTTACAAGCTTCATTTCCTAATTCAA
Ces3a	ACTGGGACCTCTTCGGTTCT	GGTGAATCTGCTGTTGCTCA
Claudin-3	CACCACTACCAGCAGTCGATGAAC	AGACTGTGTGTCGTCTGTCACCATC
Cyp2f2	AAATACCCCAAAGTGCAAGC	TGCATCTGTGTAAGGCATGG
Cyp3a11	TGAATATGAAACTTGCTCTCACTAAAA	CCTTGTCTGCTTAATTTCAGAGG
Cyp51	ATCCAGAAGCGCAGGCTGTCAA	CAGTCCGATGAGCATCCCTGAT
Ephx1	GAGTGGAGGAACTGCACACC	AGCACAGAAGCCAGGATGA
Gsta2	GAGCTTGATGCCAGCCTTCTGA	TTCTCTGGCTGCCAGGATGTAG
Hmgcr	CCTCTCTACAGTACCTGCCTTACA	CCGATCACATTCTCACAGCA
Ldlr	CAAGAGGCAGGGTCCAGA	CCAATCTGTCCAGTACATGAAGC
Nqo1	AGCGTTCGGTATTACGATCC	AGTACAATCAGGGCTCTTCTCG
Occludin	GTCCGTGAGGCCTTTTGA	GGTGCATAATGATTGGGTTTG
β-actin	CCAACCGTGAAAAGATGACC	ACCAGAGGCATACAGGGACA
