# Supplementary material for: Molecular alterations associated with pathophysiology in liver-specific ZO-1 and ZO-2 knockout mice
Source: Cell Struct Funct. 2024 Sep 26;49(2):83–99. doi: 10.1247/csf.24046 (PMC11930773; doi:10.1247/csf.24046)
Supplement: Supplementary file 1 — Supplementary Materials [file csf_49_24046_1.zip › 49_24046_Suppl_Fig_legends/49_24046_Suppl_Fig_legends.docx]

**Figure S1. Histological Analysis of Mouse Liver at 2 Weeks of Age.**

Hematoxylin and eosin staining of liver sections from WT and DKO mice at 2 weeks of age. C, central vein; P, portal vein. Scale bar: 30 μm.

**Figure S2. Analysis of oxidative stress by staining 4-HNE in Mouse Liver.**

(A) Frozen sections of the liver from WT and DKO mice were stained with the anti-4-HNE antibody conjugated with Alexa Fluor 594. Scale bar: 20 μm. (B) The fluorescence signal intensity was quantified and represented as Integrated Density using Fiji.

**Figure S3. Expression of ZO-1 and ZO-2 in E15.5 and E17.5 Mouse Embryo Livers.**

(A) Western blot data showing the protein expression levels of ZO-1 and ZO-2 in liver samples from floxed-ZO-1/ZO-2 embryos at E15.5 and E17.5, with or without Alb-Cre. (B) The quantitative analysis of the western blot data. Data are presented as mean ± standard error of the mean (n=4). Statistical significance relative to Alb-Cre (-) is indicated as **P < 0.01.
