# Supplementary figures and images for: Molecular alterations associated with pathophysiology in liver-specific ZO-1 and ZO-2 knockout mice
Source: Cell Struct Funct. 2024 Sep 26;49(2):83–99. doi: 10.1247/csf.24046 (PMC11930773; doi:10.1247/csf.24046)

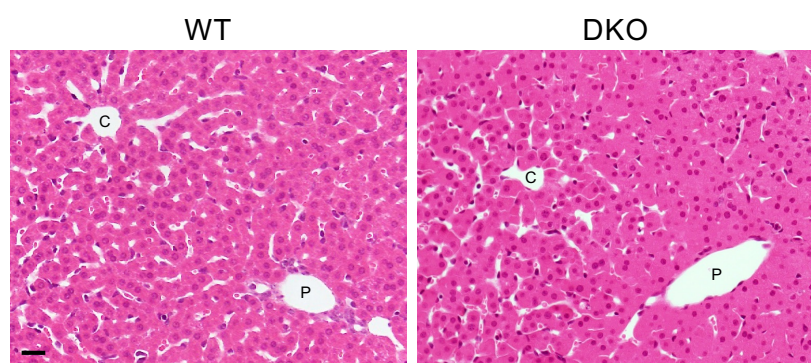

**Figure S1**

Supplement: Supplementary file 1 — Supplementary Materials [file csf_49_24046_1.zip › 49_24046_Suppl_Fig_legends/49_24046_Fig_S1.pdf]

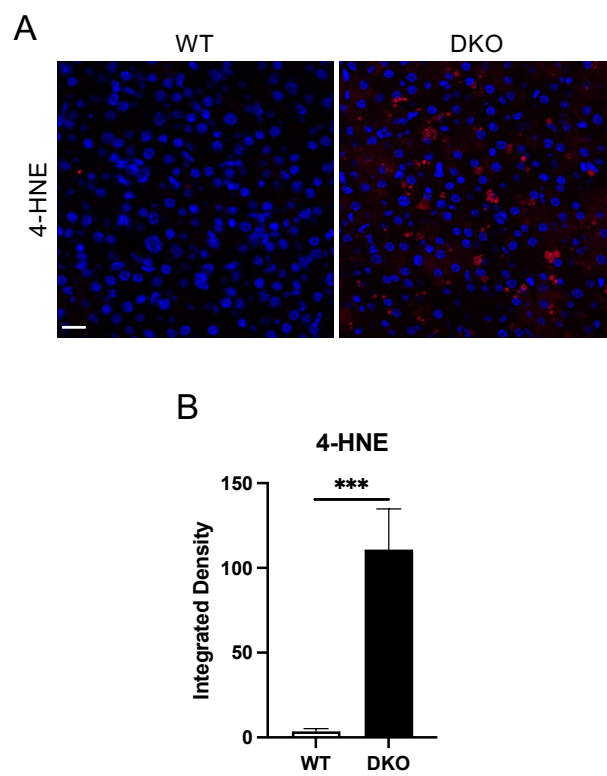

**Figure S2**

Supplement: Supplementary file 1 — Supplementary Materials [file csf_49_24046_1.zip › 49_24046_Suppl_Fig_legends/49_24046_Fig_S2.pdf]

**A**

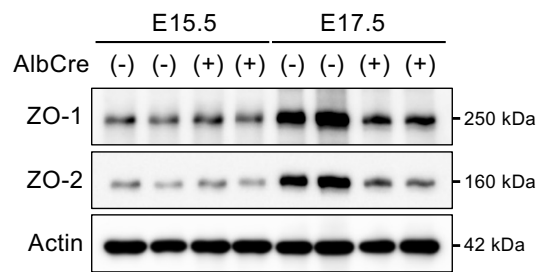

**B**

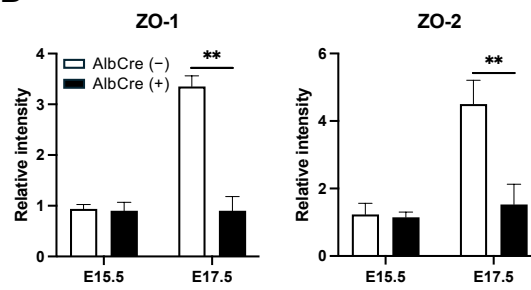

**Figure S3**

Supplement: Supplementary file 1 — Supplementary Materials [file csf_49_24046_1.zip › 49_24046_Suppl_Fig_legends/49_24046_Fig_S3.pdf]
